# The feasibility and outcome of laparoscopic vesicovaginal fistula repair as a minimally invasive technique: a retrospective descriptive monocentric study

**DOI:** 10.11604/pamj.2023.44.101.35033

**Published:** 2023-02-22

**Authors:** Ameni Bouattour, Badreddine Ben Khalifa, Sahbi Naouar, Mohamed Amri, Wael Gazzeh, Salem Braeik, Rafik El Kamel

**Affiliations:** 1 Obstetrics and Gynecology Department, *CHU* Hedi Chaker, Sfax, Tunisia,; 2 Urology Department, *CHU* Ibn El Jazzar, Kairouan, Tunisia,; 3 Faculty of Medicine of Sousse, University of Sousse, Sousse, Tunisia,; 4 Surgery Department, Centre Hospitalier Verdun Saint Mihiel, Verdun, France

**Keywords:** Iatrogenic, retrotrigonal, laparoscopic, extravesical, vesicovaginal fistula

## Abstract

**Introduction:**

vesicovaginal fistula (VVF) is the most common type of urogenital fistula. The laparoscopic approach to VVF repair offers the advantage of minimally invasive surgery with similar principles to the open trans-abdominal approach. The purpose of our study was to evaluate the transperitoneal laparoscopic approach as a minimally invasive tool for VVF repair.

**Methods:**

this was a retrospective study including 14 patients with VVF who underwent transperitoneal laparoscopic fistula repair between 2016 and 2020 in the urology department of the university hospital, Kairouan. Patients had undergone surgery at least six months after their primary gynecological surgery and were followed during 9 months after laparoscopic fistula repair. Data regarding patients' characteristics, operative data, and outcomes were gathered. The main outcome was the success rate of VVF closing and postoperative complications.

**Results:**

fourteen patients were included. The patient's mean age was 34.8±8.2years. Size of fistula varied from 0.5 to 2cm and all the VVF were supratrigonal. The mean operative time was 145±23.4 minutes with no significant blood loss. The mean hospital stay was 4±1.4 days without major complications. Regarding analgesia, paracetamol was used for the first two days to meet the analgesia needs of all patients, and morphine was used in three cases (21.4%). During follow-up, two patients were re-operated for early recurrence (14.2%) and the total success rate was 85.7% (12 patients).

**Conclusion:**

the laparoscopic repair of VVF is a safe, effective, minimally invasive procedure, and without major complications.

## Introduction

The iatrogenic vesicovaginal fistula (VVF) rate is increasing in developing countries due to increased incidence of obstetric trauma and hemostatic hysterectomies [1]. Surgical repairs of VVF most commonly performed: vaginally, abdominally, and laparoscopically. Each year, approximately half a million new cases of fistula result in obstetric complications worldwide [1]. Although the incidence of iatrogenic fistulas is on the rise, it became rare in the industrialized countries due the immense progress in surgery's techniques [2].

The approach to VVF repair is often dictated by the surgeon's preference, location or complexity of the VVF [3]. Minimally invasive techniques such as laparoscopic surgery and robotic surgery are new approaches to treating VVF [4]. Laparoscopic repair is an attractive technique and it is now a well-established modality in the management of VVF, with a number of studies demonstrating its safety, feasibility, and efficacy with a good success rate and less morbidity compared with those of open surgery [5]. These new approaches have been shown to be as effective as open surgery and with success rates varying between 90% and 100% [6,7]. We report our preliminary results concerning a minimally invasive technique for repairing VVFs using the transperitoneal laparoscopic approach.

## Methods

**Study design:** this was a retrospective descriptive study including 14 patients who operated for iatrogenic VVFs via transperitoneal laparoscopic route collected during 5 years from January 2016 to December 2020 within the urology department of the University Hospital, Kairouan. Urogenital fistula was classified as iatrogenic if the fistula developed after pelvic surgery (hysterectomy for nonobstetric causes), cesarean delivery, or cesarean hysterectomy. Data regarding patients' characteristics, operative data, and outcomes were gathered.

**Inclusion criteria:** we included Patients with iatrogenic VVF (C-section, hysterectomy); supra trigonal VVF and those operated by laparoscopic route.

**Exclusion criteria:** excluded were patients who had undergone vaginal surgery, open surgery and whose VVF was managed by simple draining.

**Figure 1:**
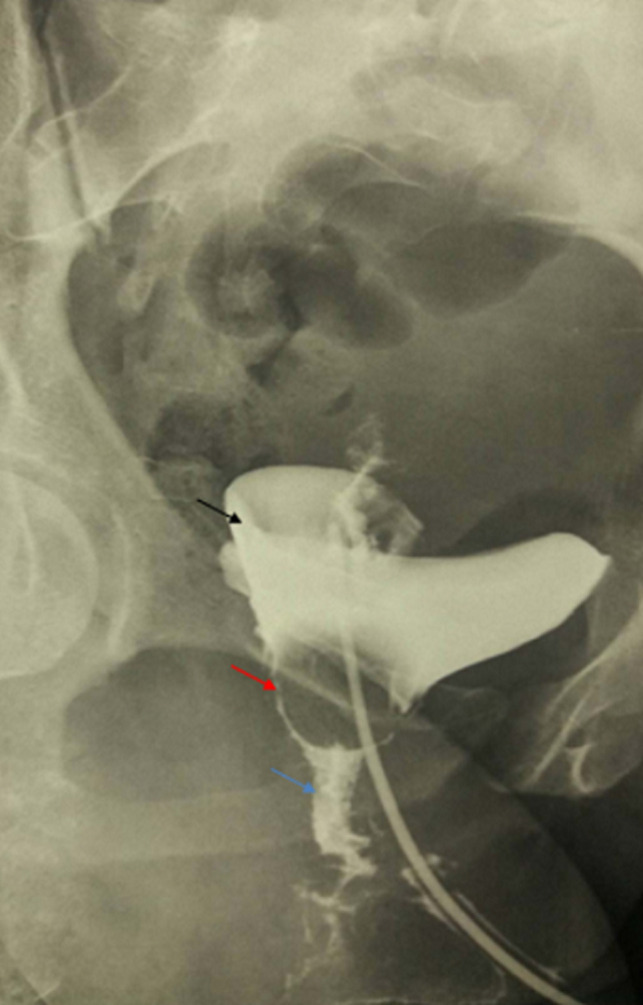
high VVF on retrograde cystography (black arrow: bladder, red arrow: vagina, blue arrow: fistula tract)

**Preoperative assessment:** all patients underwent a careful clinical examination including a gynecological examination under vaginal valves, cystoscopy and a physiological serum leak test to characterize and locate the fistula. A radiological exploration including either a computerized tomography (CT) urogram or a retrograde cystography was performed (Figure 1). The 6-month post-operative period was respected to perform the surgery on all these patients, and the operability was checked (a normal cytobacteriological examination of urine (CBEU), a normal preoperative checkup). The surgery was made by two entrained surgeons with more than twenty years of experience in urological diseases.

**Figure 2:**
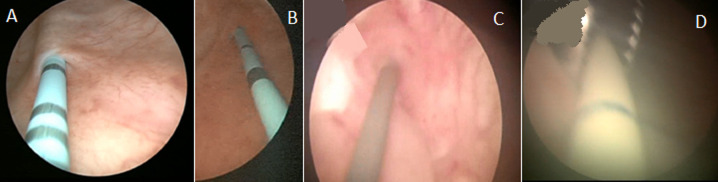
stents were inserted in both ureters to identificate ureteral orifices during the procedure; A) right ureteral orifice, B) left ureteral orifice; C,D) catheterization of fistulous path with a ureteral catheter of different color

**Surgical method:** all patients received general anesthesia and were placed in the lithotomy position. Our technique consisted of a first cystoscopy to locate the fistulous tract and catheterize it with a ureteral catheter of different color to facilitate its subsequent location (Figure 2). Then and after making the pneumoperitoneum of twelve millimeter of mercury, a 0° optical trocar is placed under the umbilicus, then two trocars of 5 millimeters and one of ten millimeters in a fan are introduced, the operator being to the left of the patient.

**Figure 3:**
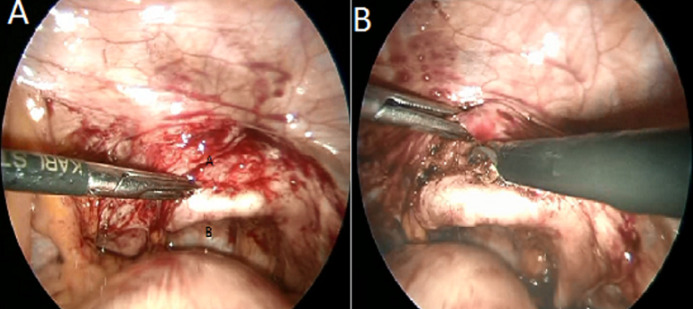
A) exposition of intervesico-vaginal space by a malleable valve putting down the vaginal fund, B) dissection of the intervesico-vaginal plane

**Figure 4:**
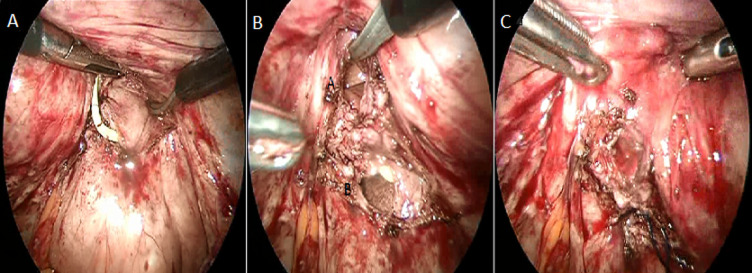
A) excision of the fistula path identified by ureteral catheter, B) section of the catheter showing the vesical and vaginal orifice, C) closure of the vagina by vicryl 2/0

The intervesico-vaginal space is exposed by a malleable valve putting down the vaginal fund (Figure 3). Therefore, it is necessary to advance in the vesicovaginal separation from back to front until meeting the fistulous path identified by the ureteral probe (Figure 4).

**Figure 5:**
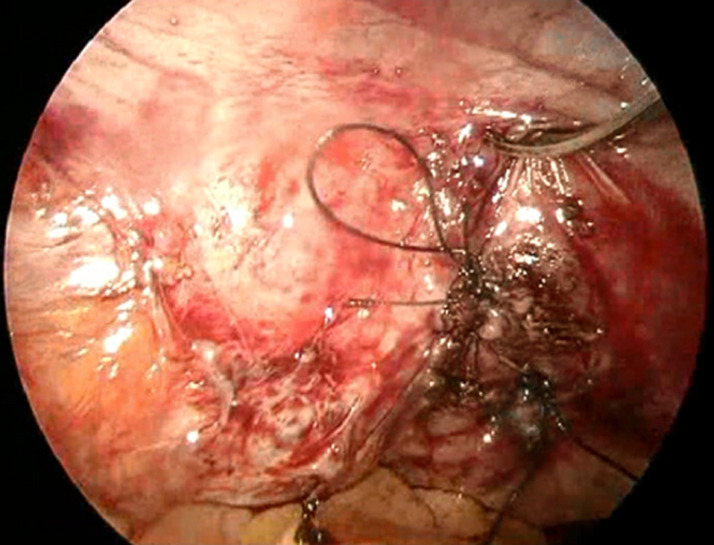
closure of the bladder by vicryl

The fistula is bypassed from the sides to find the healthy plane. A glove filled with compresses is inserted into the vagina so as not to lose the pneumoperitoneum. The edges of the bladder opening of the fistula are resected to a minimum and the suture of the bladder is carried out by an overlock of 3/0 monofilament. The vagina is closed in the same way using a 2/0 thread (Figure 5). The use of the new self-locking threads makes it easier to maintain tension while suturing. After that, omentum fat or a peritoneal flap is interposed which is fixed as distally as possible in the vesicovaginal separation plane.

Finally, a drainage of the intervesico-vaginal space is put in place and kept on average for 3 days. For the ureteral catheters, we remove them within one to two weeks. A silicone trans-urethral catheter is put in place and kept for 2 to 3 weeks until a cystography is performed to check the bladder seal. Another control by cystography was carried out three months after the operation for all our patients. A monthly follow-up was practiced until 9 months after surgery.

**Data collection and outcome:** patient data were collected on age, parity, type and etiology of fistula. The main outcome was the success rate of VVF closing and postoperative complications.

**Ethical considerations:** ethical approval was obtained from the Sousse University Institute of Medical Sciences Institutional Review Board. The study was discussed with recruited patients and informed consent was provided.

**Statistical analysis:** SPSS (IBM SPSS, version 21, Armonk, IBM Corp, Armonk, NY) was utilized for statistical analysis. Measures of central tendency (average) and dispersion (standard deviation) were presented for quantitative variables and absolute frequencies and percentages were presented for categorical variables.

## Results

The patient's characteristics are summarized in Table 1. The main age was 34.8±8.2 years with extremes ranging from 24 to 51 years. Most of them (78%) were from urban areas. The main symptom that led us to discover the VVF was the permanent leakage of urine, observed in all our patients. The time from previous operation to laparoscopic repair was 6 months in all cases. The main cases of VVF were followed abdominal hysterectomy in 12 cases and cesarean section in 2 cases. All fistulas were located in the retro trigonal region and their sizes varied between 0.5 and 2cm. The mean operative time was 145±23.4 minutes (range 110 min -180 min) including the introduction of trocars. Blood loss was minimal and none of our patients required a blood transfusion.

**Table 1:** demographic and clinical characteristics

Characteristic	Value
Age(year)	34.8±8.2
Residence (urban/rural)	11 (78%)
Time to diagnose the disease(month)	1-5
**Etiology (n, %)**	
Abdominal hysterectomy	12 (85%)
Cesarean section	2 (15%)
Size of fistula(mm)	8 (5-20)
**Fistula site, n (%)**	
Retro trigonal	14 (100%)
Infra trigonal	0 (0)

Systematic drainage of the intervesico-vaginal space was kept for an average of 3 days (2 - 6 days). The foley catheter was kept on average for 13 days with extremes ranging from 10 to 21 days. A cystography was performed in all operated patients showing bladder integrity. The mean hospital stay was 4±1.4 days (range 2 - 7 days). The analgesic used postoperatively was Paracetamol IV for all patients at a rate of 1000mg every 6 hours for an average duration of 2 days; we needed morphine's for three patients. The results were satisfactory for twelve patients with a follow-up of 9 months. Two patients had a second repair by conventional surgery for early recurrence (2 months) (Table 2).

**Table 2:** intraoperative and postoperative data

variables	Value
Operative time (min)	145±23.4
Need for transfusion (n)	0
Hospital stays (days)	4±1.4
**Use of analgesics**	
Paracetamol	14 (100)
Morphine's	3 (21.5)
Time of removal drainage (days)	3 (2-6)
Duration of bladder catheterization (days)	13 (10-21)
Success after 9 months	12 (85%)

## Discussion

Our results showed that repair of VVFs with laparoscopic surgery is an effective and safe procedure, with a high success rate, and is an alternative in the treatment of iatrogenic VVF. Previously, the causes of urogenital fistula in developing countries were not linked to iatrogenic causes. Iatrogenic fistulas, on the other hand, appear to be on the rise in recent years [8]. A retrospective study performed in 11 countries, mostly in Africa, between 1994 and 2012 found that iatrogenic mistakes caused 13.2% of urogenital fistula cases [9]. According to a recent Ethiopian study, 24.6% of 2500 fistula cases were attributed to surgical causes, specifically cesarean delivery, and hysterectomy [10]. A recent African study found that despite the strong association between obstetric fistula and prolonged, obstructed labor, more than a quarter of women with fistula after cesarean birth had injuries due to surgical complications rather than pressure necrosis [11].

The gradual increase in iatrogenic fistula is a warning sign about the quality of health care and training systems. Although surgical training may provide a solution, health professionals may lack the necessary practical experience to deal with complicated deliveries and surgical procedures. As a result, advanced training is required for improved decision-making and surgical skills in both obstetric and gynecologic management, particularly for safe cesarean delivery and hysterectomy. Furthermore, despite the declining trend in obstetric fistula, steps must be taken to improve health services in areas with limited access [9]. These measures will eventually result in a better healthcare system and a lower rate of fistula development [12]. Minimally invasive techniques such as laparoscopic surgery and robotic surgery are new approaches to VVF treatment that have resulted in decreased morbidity and faster recovery.

In 1998, von Theobold *et al*. were among the first to report a case of laparoscopic extravesical repair of VVF [12]. Depending on the used technique, closing the bladder opening only without closing the vagina and interposing an epiploic sleeve is sufficient. A few years later, Miklos *et al*. described a new approach which consisted of a bladder closure in two planes, a vaginal closure as well as the interposition of healthy vascularized tissues between these two planes [13,14]. There is no significant difference between the results obtained by the O'connor transvesical approach and the extra bladder approach. For this, the extravesical technique had more advantages in terms of respecting the anatomical integrity of the bladder [8,5,11]. The interposition of an omentum sleeve or peritoneal flap is recommended by most authors, which is explained not only by the presence of a new sealing plane but also by the improvement of vascularization and lymphatic drainage guaranteeing a better healing [12,13,15].

Miklos *et al*. performed a systematic review regarding laparoscopic and robotic-assisted vesicovaginal fistula repair. In this review, the overall success rate of laparoscopic VVF repair was 80% to 100%, with a follow-up period of 1 to 74 months. The success rate of transvesical and extravesical techniques were 95.89% and 98.04%, respectively [8]. Successful treatment of VVF is highly dependent on the surgeon's experience, tissue conditions around fistulae, and adequate postoperative urinary drainage [12,15,16]. In our study, we used a minimally invasive technique with a high rate of success (85%). However, the main limitation is the small number of patients operated on. These are preliminary results from the start of the experiment with a success rate comparable to most studies.

The current study had several limitations. The retrospective nature and the small number of participants limited us to performing a robust statistical analysis. We did not use another surgical procedure to assess the efficacy of our procedure. A prospective multicentric comparative study with a large number and extended the postoperative follow-up is recommended to confirm our result.

## Conclusion

Our study improved that repair of VVFs with laparoscopic surgery is an effective and safe procedure, with a high success rate, and is an alternative in the treatment of VVF.

### What is known about this topic

- *Laparoscopic repair of vesicovaginal fistula (VVF) may be a feasible, effective, and mini-invasive approach for the treatment of VVF;*
- *Because of the limited number of patients in different studies published in literature, a larger number of cases are needed to establish the effectiveness of this technique in the long term*.

### What this study adds

- *This study reports the experience of our department in the laparoscopic repair of VVF;*
- *It is the first study published about this field in our country*.
